# Randomized Controlled Study Evaluating Efficiency of Low Intensity Transcranial Direct Current Stimulation (tDCS) for Dyspnea Relief in Mechanically Ventilated COVID-19 Patients in ICU: The tDCS-DYSP-COVID Protocol

**DOI:** 10.3389/fmed.2020.00372

**Published:** 2020-06-26

**Authors:** Eric Azabou, Guillaume Bao, Nicholas Heming, Rania Bounab, Pierre Moine, Sylvain Chevallier, Sylvie Chevret, Matthieu Resche-Rigon, Shidaps Siami, Tarek Sharshar, Frederic Lofaso, Djillali Annane

**Affiliations:** ^1^Clinical Neurophysiology and Neuromodulation Unit, Departments of Physiology and Critical Care Medicine, Raymond Poincaré Hospital, Assistance Publique-Hôpitaux de Paris (AP-HP), Inserm UMR 1173, Infection and Inflammation (2I), University of Versailles Saint-Quentin en Yvelines (UVSQ), Paris-Saclay University, Paris, France; ^2^General Intensive Care Unit-Assistance Publique Hôpitaux de Paris, Raymond Poincaré Hospital, Assistance Publique-Hôpitaux de Paris (AP-HP), Inserm UMR 1173, Infection and Inflammation (2I), University of Versailles Saint-Quentin en Yvelines (UVSQ), Paris-Saclay University, Paris, France; ^3^Versailles Engineering Systems Laboratory (LISV), University of Versailles Saint-Quentin en Yvelines (UVSQ), Paris-Saclay University, Velizy, France; ^4^Service de Biostatistique et Information Médicale, AP-HP Hôpital Saint Louis, Paris, France; ^5^Inserm U1153 CRESS, Epidemiology and Clinical Statistics for Tumor, Respiratory, and Resuscitation Assessments (ECSTRRA) Team, Paris, France; ^6^Université Paris 7 Diderot, Sorbonne Paris Cité, Paris, France; ^7^Critical Care Medicine Unit, CH Etampes-Dourdan, Etampes, France; ^8^Department of Neuro-Intensive Care Medicine, Sainte-Anne Hospital, Paris-Descartes University, Paris, France; ^9^Laboratory of Human Histopathology and Animal Models, Institut Pasteur, Paris, France

**Keywords:** COVID-19, acute respiratory distress syndrome (ARDS), tDCS, dyspnea relief, brain, neuromodulation, mechanical ventilation, ICU

## Abstract

The severe respiratory distress syndrome linked to the new coronavirus disease (COVID-19) includes unbearable dyspneic suffering which contributes to the deterioration of the prognosis of patients in intensive care unit (ICU). Patients are put on mechanical ventilation to reduce respiratory suffering and preserve life. Despite this mechanical ventilation, most patients continue to suffer from dyspnea. Dyspnea is a major source of suffering in intensive care and one of the main factors that affect the prognosis of patients. The development of innovative methods for its management, especially non-drug management is more than necessary. In recent years, numerous studies have shown that transcranial direct current stimulation (tDCS) could modulate the perception of acute or chronic pain. In the other hand, it has been shown that the brain zones activated during pain and dyspnea are close and/or superimposed, suggesting that brain structures involved in the integration of aversive emotional component are shared by these two complex sensory experiences. Therefore, it can be hypothesized that stimulation by tDCS with regard to the areas which, in the case of pain have activated one or more of these brain structures, may also have an effect on dyspnea. In addition, our team recently demonstrated that the application of tDCS on the primary cortical motor area can modulate the excitability of the respiratory neurological pathways. Indeed, tDCS in anodal or cathodal modality reduced the excitability of the diaphragmatic cortico-spinal pathways in healthy subjects. We therefore hypothesized that tDCS could relieve dyspnea in COVID-19 patients under mechanical ventilation in ICU. This study was designed to evaluate effects of two modalities of tDCS (anodal and cathodal) vs. placebo, on the relief of dyspnea in COVID-19 patients requiring mechanical ventilation in ICU.

**Trial Registration:** This protocol is derived from the tDCS-DYSP-REA project registered on ClinicalTrials.gov NCT03640455. It will however be registered under its own NCT number.

## Introduction

Dyspnea is a “symptom” common to various ailments and pathologies such as sepsis, asthma attack, intoxication, severe metabolic disorders known for their association with acute respiratory distress syndrome (ARDS) (1–3). More than half of patients admitted to intensive care for septic shock have an ARDS (4). Coronavirus disease 19 (COVID-19) caused by severe acute respiratory syndrome coronavirus (SARS-CoV-2) presents in its severe forms a severe respiratory distress syndrome requiring patients to be put on mechanical ventilation in intensive care (5–8). This respiratory suffering has a dyspneic component, which often reaches unbearable limits and constitutes a major factor in altering the clinical state and the prognosis of patients (6, 9). Dyspnea usually persists despite adequate treatment of the underlying pathology, or sometimes worsens after it has normalized (10–13). This phenomenon of perceptual dysfunction (exaggerated, persistent perception) is linked to changes in cortical excitability due to neuronal plasticity (14). The pathophysiologic mechanisms of dyspnea are quite complex, but are beginning to be better understood (15). The dysfunctions can occur around the thoracic mechanics, the respiratory muscles and blood gases. They may also occur within the neurological and neurobiological structures ensuring the central integration. In particular there are afferents to the cortex which are compared with the motor pathways via corollary discharge (16, 17). Poor adaptation of the ventilator is also a main cause of dyspnea (3). Dyspnea appears when the respiratory work becomes excessive, in particular when the abnormalities of the respiratory mechanics increases the respiratory work, or when the capacities of ventilation are lower than the needs for the organization (18–21). The length-tension ratio of the respiratory muscle fibers, the numerous neurochemical receptors located in the chest wall, the lungs, the airways, the vascular walls, and also in the cerebral centers of respiration are all actors involved in these mechanisms. The brain correlates of respiratory discomfort have been described by several recent works (2, 22, 23). Analogies are drawn between the pathophysiology of dyspnea sensations and that of pain (14, 24, 25). It is a multidimensional experience resulting from a complex central integration of the interaction between multiple factors, physiological, social, and environmental (26). However, despite the appropriate treatment of the recognized or suspected underlying cause and normalization of the blood gazes, dyspnea is often insufficiently relieved and therefore requires—as with pain—specific treatments for this symptom (1). This applies particularly to the hyperventilation syndrome which often persists after the normalization of the underlying functional impairment, and even more so to “medically unexplained” or “psychogenic” dyspnea (11, 17). Recent years, numerous studies have shown that transcranial direct current stimulation tDCS was able to modulate, the perception of acute (27–29) or chronic pain (30–32) which raised hopes of being able to use this technique in the treatment of refractory pain by conventional therapeutic means. Studies in functional brain imaging have been able to show that the effects of this cortical stimulation—in terms of brain activity—were not limited to the cortical zone next to the stimulation electrode (33) but involved a whole set of brain structures (some of which are quite far from the stimulation site) including the anterior cingulum gyrus, the prefrontal cortex, the thalamus, the brainstem, and even the spinal cord (34, 35). While the role of some of these structures in the central integration of pain is currently well-established, they are likely to be also involved in the central integration of dyspnea. Indeed, functional imaging studies on dyspnea (36), in particular one which jointly assessed pain and dyspnea (37) have highlighted activation zones that are close or even superimposed for pain and dyspnea, probably corresponding to brain structures involved in the integration of the aversive emotional component shared by these two complex sensory experiences. Therefore, it can be hypothesized that stimulation by tDCS with regard to the areas which, in the case of pain activated one or more of these cerebral structures could also have an effect on dyspnea. In addition, our team recently demonstrated that the application of tDCS on the primary cortical motor area can modulate the excitability of the respiratory neurological pathways. Indeed, tDCS in anodal or cathodal modality allowed a reduction in the excitability of the diaphragmatic cortico- spinal pathways in healthy subjects (38).

We hypothesized that tDCS could relieve dyspnea in COVID-19 patient requiring mechanical ventilation in ICU (39, 40). We designed this project to assess the effectiveness of tDCS on the relief of dyspnea in COVID-19 patients requiring mechanical ventilation in ICU.

## Materials and Methods

### Study Population

This study will enroll 63 (3 groups of 21) COVID-19 patients, admitted in ICU with ARDS requiring mechanical ventilator for at least 24 h, and having significant dyspnea (dyspnea level ≥4 on the A1 subscale of the Multidimensional Dyspnea Profile) (1, 41).

#### Inclusion Criteria

- Adult patient, hospitalized in intensive care for COVID-19, having required mechanical ventilation for at least 24 h.
- Not sedated or having a good awakening (Richmond Agitation Score- Sedation Scale (RASS)> −3 at the time of inclusion (42) within 48 h of stopping sedation.
- Able to answer yes or no to simple questions.
- Having significant dyspnea (level≥ 4) on the A1 scale of the Multidimensional Profile of Dyspnea (MPD-A1≥ 4) (1, 41).
- Signature of informed consent by the patient or his family member.

#### Exclusion Criteria

- Patient under guardianship,
- Wake up delay, coma (GCS≤ 8), or severe agitation.
- Chronic respiratory pathology.
- Medical history of respiratory, neuromuscular, or neuro-sensorial handicap (auditory or visual) pathology.
- Language barrier, refusal to participate in the study or to sign the informed consent,
- Pregnant or lactating woman,
- No affiliation to a social security scheme.

### Objectives

#### Main Objective

The main objective of this study was to determine whether tDCS allowed a significant reduction in dyspnea, measured by the A1 subscale of the multidimensional profile of dyspnea (MPD-A1), in patients admitted to intensive care for COVID-19 placed on mechanical ventilation and suffering dyspnea.

#### Secondary Objectives

- To evaluate the effect of tDCS on the different components of dyspnea using the other subscales of the multidimensional profile of dyspnea “MPD”: sensory (MPD-QS) and emotional (MPD-A2) subscales.
- To determine if tDCS also allowed a significant reduction in dyspnea measured by the IC-RDOS scale (intensive care respiratory distress observation scale) (43).
- To investigate the presence of pre-inspiratory potentials (PPI) on the EEG in this patient population and determine the effect of tDCS on these PPIs in patients who may have them.
- To evaluate the effect of tDCS on respiratory parameters: mouth pressure (amplitude of variation), PetCO2, tidal volume (VT), and respiratory rate (F) as well as ventilation/minute (calculated from VT and F).
- To evaluate the impact of the possible relief of dyspnea by tDCS on the patient's close outcome during the 28 days following inclusion: mortality in intensive care, in hospital on D28, the cumulative incidence of delirium and its duration until D28, the cumulative incidence of mechanical ventilation, the failure to wean from mechanical ventilation on D28, and the length of stay in intensive care.

### Evaluation Criteria

#### Primary Endpoint

- Measurement of the differential of the score on the A1 subscale of the Multidimensional Dyspnea Profile (MPD-A1) (from 0 to 10): between before and after the use of tDCS. This primary judgment criterion will be assessed by an independent, blind observer. The differential will be measured between 30 min before the procedure and 30 min after.

#### Secondary Endpoints

- Differentials of the MDP-QS and MDP-A2 subscales of the Multidimensional Profile of Dyspnea measured between before and after tDCS: in order to assess the effect of tDCS on the different components of dyspnea: sensory (MPD-QS) and emotional qualifiers (MPD-A2 subscales).
- Differential in the IC-RDOS (intensive care respiratory distress observation scale) scale between before and after the use of tDCS. A significant reduction in this score after the use of tDCS will translate into a reduction in respiratory discomfort, especially dyspnea.
- Pre-inspiratory potentials (PPI) assessment: the possible presence of PPI on the EEG in this patient population could be a marker of respiratory suffering, and a possible disappearance of PPI after the use of tDCS could be interpreted as a relieving effect on breathing difficulty.
- The respiratory parameters measurement: mouth pressure (amplitude of variation), PetCO2, tidal volume (VT), and respiratory rate (F) as well as ventilation/minute (calculated from VT and F), between before and after use of tDCS.
- Evaluation of the impact of tDCS on the outcome of patients during the 28 days following inclusion:
  - Death by D28 in intensive care and in the hospital,
  - Cumulative incidence of delirium and its duration (CAM-ICU scale) (44).
  - Proportion of patients with mechanical ventilator dependance beyond D28.
  - Cumulative incidence of mechanical ventilation on D28,
  - The duration of the resuscitation stay.

### Description of the Evaluation Parameters and Measurement Techniques

#### The Multidimensional Dyspnea Profile (MPD)

The A1 subscale of the multidimensional profile of dyspnea allows to diagnose dyspnea and to rate its intensity. This score is the equivalent of the visual analog scale. A score of four is considered the lower limit for moderate dyspnea (3). The QS (sensory qualifiers) and A2 (emotional) subscales allow better specifying and defining the type of components that characterize each patient's dyspnea (1, 41). These different subscales of the multidimensional profile of dyspnea will be performed before the start of tDCS, then after the end of tDCS for each patient.

#### The IC-RDOS (Intensive Care Respiratory Distress Observation Scale) Score

The IC-RDOS scale is derived from the respiratory distress observation scale (RDOS). It is composed of the five items (heart rate, use of the neck muscles during inspiration, paradoxical abdominal movement, facial expression of fear, and additional oxygen) and is validated to serve as tools for objective and reliable evaluation of dyspnea in resuscitation patients (43) and could therefore be used as an alternative to psychometric scales to assess dyspnea in patients who are unable to communicate verbally.

#### The Pre-inspiratory Evoked Potentials (PPI)

PPIs are slow brain waves generated during the milliseconds preceding the start of inspiration in healthy subjects in a situation of voluntary or forced breathing, and in patients suffering from respiratory discomfort: COPD, asthma, respiratory distress, Ondine (45–50). These potentials are absent in the case of spontaneous breathing in healthy subjects and disappear in patients as soon as the respiratory discomfort is removed. EEG signal was synchronously recorded with the respiratory flow and pressure signals using a Nihon Kohden France manufactured EEG-9100J/K, digital EEG system. Scalp electrodes were placed according to the conventional “10–20” topographic system, via a 19-electrodes cap installed after rubbing and cleaning with alcohol and application of a conductive gel. The ground electrode was positioned at Fpz. The EEG traces are then divided into 3 s sections centered on the start of inspiration (from 2.5 s before the start of inspiration until 0.5 s after the start of inspiration). At least 40–50 EEG samples are required. These 40–50 EEG samples thus cut are then averaged to objectify the PPI. This step of analysis and processing of signals (sampling and averaging) is done automatically using EEG software. The presence or not of the PPI recorded during the 30 min preceding the start of stimulation with tDCS will be recorded, as well as during the 30 min following the cessation of tDCS.

#### The Respiratory Parameters

The subject being connected to the ventilator, by measuring devices corresponding to a series connection which comprises—downstream of the subject—a device equipped with a CO2 sensor and a pneumotachograph, the pressure sensor of which also makes it possible to measure the mouth pressure (Pm) (NICO2 sensor combined CO2 adult flow Novametrix Nico).

The oxygen saturation will be determined with your finger using a pulse oximeter (Novametrix Oxymeter). The acquisition of all of these respiratory signals (Pm, instantaneous flow rate, expired CO2 and SatO2) is carried out during a period of 15 min before the introduction of tDCS, then again for 15 min after the end of tDCS.

The following respiratory parameters will be precisely measured and calculated:

- The pressure measured at the mouth (Pm), the amplitude of variation of which (aPm), gives an indirect but fairly practical reflection of the additional respiratory effort, which, in the case of dyspnea linked to laden breathing is one of the parameters that is best correlated with its intensity.
- PETCO2: the partial pressure of CO2 at the end of expiration: by being (in the ideal case), a reflection of capnia, the increase of this being another mechanism inducing dyspnea with a strong unpleasant connotation (air hunger) especially in a context where ventilation is forced to a level lower than that which would have been chosen spontaneously. Thus, the measurement of aPm and PetCO2 will allow us to assess an equivalent of physical stimulus for each of the two types of dyspnea and to assess the relationship of these with the intensity of the dyspnea. In addition, in the case of aPm, it will provide an index of the motor response to the loads.
- Tidal volume and respiratory rate as well as ventilation/minute (calculated from the instantaneous flow signal) will provide us with an interesting insight into the adaptation of breathing to the physiological mechanisms underlying dyspnea.

#### The CAM-ICU Scale

The confusion assessment method for the intensive care unit (CAM-ICU) will be used for detection and monitoring of delirium during the 28 days of follow-up after inclusion (44).

### Experimental Design

#### Randomization

After verification of the inclusion and exclusion criteria, the patients will be prospectively and randomly included in three groups of 21 patients, depending on the type of tDCS treatment received: anodal tDCS group, cathodal tDCS group, and placebo tDCS group. The tDCS will be applied up on the cortical representation zone of the primary motor and left pre-motor cortex for 30 min; intensity 2 mA (in anodal, cathodal, or placebo modality). The patient will be blinded from the randomization arm. Randomization will be performed on a dedicated and secure specific website (Cleanweb). Randomization will be carried out in a 1: 1: 1 ratio with permutation blocks of size unknown to the investigators.

#### Description of the Acts Performed and Devices Used

This is a clinical, interventional, bi-centric, randomized, single-blind, 3-arm trial, including a placebo-controlled arm, and 2 experimental arms, evaluating the effectiveness of a medical device for therapeutic purposes (tDCS) with 63 (3 groups of 21) patients on mechanical ventilation in intensive care with dyspnea. The primary endpoint will be assessed by an independent, blind observer. Transcranial stimulation will be delivered using a medical certified “Starstim 8” brain stimulator controlled via Bluetooth using a laptop computer (Neuroelectrics, Barcelona, Spain), Stimulation will delivered through traditional 5 × 7 cm rectangular sponge electrodes, with a contact area of 35 cm^2^ (Sponstim, Neuroelectrics, Barcelona, Spain). The tDCS will be applied upon the left hemisphere because of the functional dominance of this hemisphere in humans and in accordance with previous studies having evaluated the effect of tDCS on pain and the respiratory tract (27, 28, 32, 38, 51). As described in our previous work (38), two identical, rectangular, saline-soaked electrodes, each 7 cm long and 5 cm wide (35 cm^2^), were secured to the scalp. For anodal tDCS, the anode will be placed over the left diaphragmatic primary motor cortex (4 cm lateral to the midline and 1 cm anterior to the binaural line) and the cathode will be placed above the right orbit. These positions will be switched to obtain cathodal tDCS. For both anodal and cathodal tDCS, intensity will be 2 mA and the duration 30 min. The current density used will be 0.057 mA/cm^2^, which has been proven to be safe (52–55). For the sham condition (placebo tDCS), intensity was also 2 mA but duration was only 2 min. Nitsche and Paulus reported in 2000 that at least 3 min of tDCS was necessary to induce after-effects (31).

#### Measurements of the Parameters Evaluated

**Figure 1:**
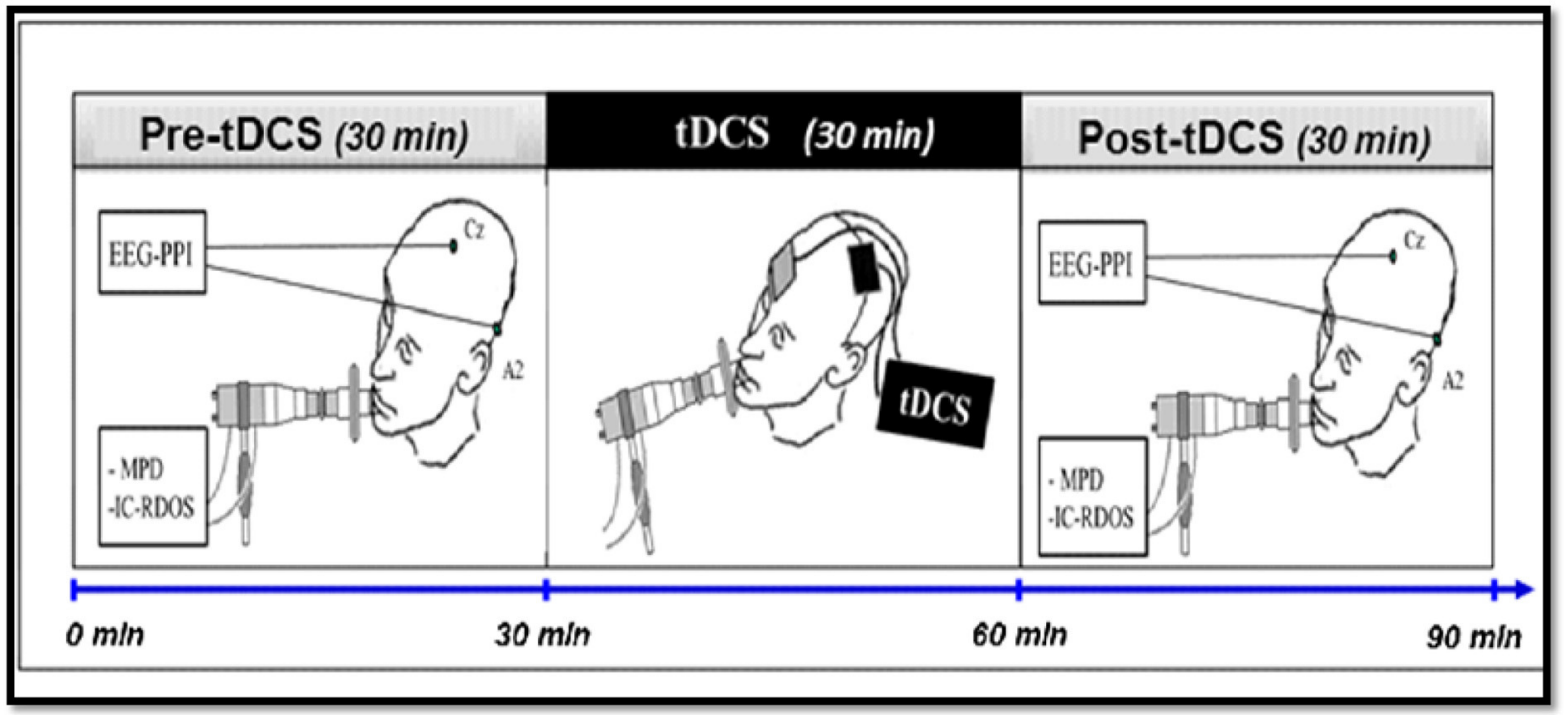
Diagram of the experimental procedure. The practical implementation of the protocol consists of a single session of ~1 h 30 min and will include the three stages. After inclusion and randomization, the set of parameters studied will be measured for each patient, for 30 min before using tDCS, then for 30 min after stopping tDCS. The tDCS will be applied for 30 min to the cortical representation area of the primary motor area and the supplementary left motor area. tDCS, Transcranial stimulation with 2 mA intensity current in anodal, cathodal or placebo polarity, applied to the cortical representation area of the left primary and pre-motor areas; EEG-PPI, EEG to measure the Pre-Inspiratory Potentials; MPD, Scales of the Multidimensional Profile of Dyspnea; IC-RDOS, Scales of the Intensive Care Respiratory Distress Observation Scale.

The parameters studied (in particular those used to calculate the main judgment criterion) will be measured during the 30 min preceding (pre) and the 30 min following (post) the use of tDCS in each of the three different experimental conditions (anodal tDCS, cathodal tDCS, and placebo tDCS). The placebo tDCS condition constitutes the control condition (fictitious stimulation: absence of delivered current (sham), therefore acting as placebo. The Figure 1 represents a diagram of the course of the experimental procedure.

#### Side-Effects and Adverse Effects Assessment

Side-effects and adverse effects associated with the tDCS during the course of the trial will be assessed using the adverse effects questionnaire proposed by Brunoni et al. for tDCS studies in order to improve systematic reporting of tDCS-related adverse effects (56).

### Research Calendar

The total duration planned for the study is 12 months. The total duration of participation for each patient is 28 days; because each patient will be followed up for a period of 28 days after inclusion in order to collect the evolution data. The Table 1 summarizes the research chronology.

**Table 1:** Summary of the research chronology.

**Actions**	**Day 0 (inclusion, before tDCS)**	**Day 0 (after tDCS)**	**Day 1 to Day 27**	**Day 28 (after tDCS)**
information	*X*			
Informed written consent	*X*			
Background	*X*			
SAPS-II score	*X*			
SOFA score	*X*			
Clinical and neurological examination	*X*	*X*	*X*	*X*
Glasgow score	*X*	*X*	*X*	*X*
RASS score	*X*	*X*	*X*	*X*
FOUR score	*X*	*X*	*X*	*X*
CAM-ICU score	*X*	*X*	*X*	*X*
Ventilation status	*X*	*X*	*X*	*X*
Ventilation weaning status (in progress, successful, failed)			*X*	*X*
Multidimensional dyspnea profile	*X*	*X*		*X*
IC-RDOS scale	*X*	*X*		*X*
EEG for PPI assessment	*X*	*X*		*X*
Respiratory Function Evaluation: PaO2, PaCO2, Mouth pressure, PetCO2, Tidal volume (VT), Respiratory frequency (F), Ventilation/minute (calculated from VT and F)	*X*	*X*		*X*

#### Selection and Inclusion Visit

Inclusion will be made when all the inclusion and non-inclusion criteria are verified and the patient has given informed consent to participate in the study. During inclusion and before the start of the single session of the protocol, the following clinical, drug, and other co- variable data will be collected. These data are in principle systematically measured in intensive care patients.

Demographic data (age and sex), the reason for admission; medical history (neurological, respiratory, cardiological) initial severity by the SAPS-II score (57), the number of organ failures by the SOFA score (58), neurological assessment scores (Glasgow, FOUR score) (59); CAM-ICU (44) and the RASS score (to assess depth of sedation (42). The determinants of secondary cerebral aggression of systemic origin: body temperature, blood pressure, PaO2, PaCO2, natremia, glycemia. The neurological examination which includes: examination of the cranial pairs (spontaneous eye movements, pupil size, photo-motor reflex, oculo-cephalogyr reflex, corneal reflex, reaction to Pierre Marie-Foix's maneuver, cough reflex), the search for archaic reflexes (corneo- mandibular, palmo- mental, yawning, chewing, grasping), osteo-tendinous, and plantar skin reflexes.

The practical implementation of the protocol will consist of a single session of ~1 h 30 m and will include the three stages described in Figure 1. After inclusion and randomization, the set of parameters studied will be measured for each patient, for 30 min before using tDCS, then for 30 min after stopping tDCS. The tDCS will be applied for 30 min to the cortical representation area of the primary motor area and the supplementary left motor area.

#### Research Follow-Up Visits

The patients will then be followed for a period of 28 days after inclusion in order to collect the evolution data including: Death, measurement of delirium (CAM-ICU scale) until D28; ventilation status (spontaneous or mechanical) and withdrawal (in progress, successful, failure) until D28, the cumulative incidence of mechanical ventilation, and the length of stay in intensive care.

#### End of Research Visit

The last visit made on D28 for patients for patients who survived to this date, will be identical to previous visits.

### Statistical Analysis

#### Descriptive Analysis

A descriptive analysis of inclusions and monitoring of the protocol will be carried out. The main analysis will be carried out according to the intention to treat principle. Only patients who have withdrawn their consent can be excluded from the analysis. Patients who have decided to discontinue the management planned for the trial, lost to follow-up, or discontinued the trial will be included in the analysis. In general, the quantitative variables will be described by their median and their first and their third quartiles and the qualitative variables will be described by the frequencies of the modalities and the associated percentages. The epidemiological and clinical characteristics of the patients at inclusion will be described by group, without statistical tests being carried out. The protocol violations, the causes of abandonment and loss of sight and the characteristics of these patients will be detailed.

#### Primary Judgment Criterion

The studied parameters will be measured during the 30 min before (pre) and 30 min following (post) the use of tDCS in all three conditions (tDCS anodal, tDCS cathodal, and tDCS placebo). The placebo tDCS condition is the control condition (fictitious stimulation). The different measures of the judgment criteria will be carried out by one of the investigating doctors blinded in the randomization arm. In order to test the effect of tDCS, the judgment criteria will be compared according to the different experimental conditions (anodal tDCS, vs. cathodal, vs. placebo tDCS). Each experimental arm will be compared to the placebo group at risk 0.025 using Students *t*-test and applying Bonferroni correction if needed.

#### Secondary Judgment Criteria

- Differentials of the MDP-QS and MDP- A2 subscales of the Multidimensional Profile of Dyspnea measured between before and after tDCS, in order to assess the effect of tDCS on the different components of dyspnea: sensory (MPD-QS) and emotional qualifiers (MPD-A2 subscales). Each experimental arm will be compared to the placebo group at risk 0.05 using a Student test. If the two experimental arms are greater than the placebo arm, they will be compared to each other at risk 0.05.
- Differential in the IC-RDOS (intensive care respiratory distress observation scale) scale: between before and after the use of tDCS. A significant reduction in this score after the use of tDCS will translate into a reduction in respiratory discomfort, especially dyspnea. Each experimental arm will be compared to the placebo group at risk 0.05 using a Student test. If the two experimental arms are greater than the placebo arm, they will be compared to each other at risk 0.05.
- Pre-inspiratory potentials (PPI): the possible presence of PPI on the EEG in this patients population could be a marker of respiratory suffering, and a possible disappearance of PPI after the use of tDCS could be interpreted as a relieving effect on breathing difficulty. Each experimental arm will be compared to the placebo group at 0.05 risk using a Fisher test. If the two experimental arms are greater than the placebo arm, they will be compared to each other at risk 0.05.
- The respiratory parameters: mouth pressure (amplitude of variation), PetCO2, tidal volume (VT), and respiratory rate (F) as well as ventilation/minute (calculated from VT and F). The comparisons of each of these parameters will be carried out. Each experimental arm will be compared to the placebo group at risk 0.05 using a Student test. If the two experimental arms are greater than the placebo arm, they will be compared to each other at risk 0.05.
- Evaluation of the impact of tDCS on the patient's future outcome during the 28 days following inclusion:
  - Death on D28 in intensive care and in the hospital: Each experimental arm will be compared to the placebo group at 0.05 risk using a Fisher test. If the two experimental arms are greater than the placebo arm, they will be compared to each other at risk 0.05. Kaplan-Meir curves for death.
  - Cumulative incidence of delirium and its duration (CAM-ICU scale): The cumulative incidence estimates will be made using the gray method and compared according to the previous procedure using a gray test.
  - Proportion of patients who failed to withdraw from mechanical ventilation on D28: Each experimental arm will be compared to the placebo group at 0.05 risk using a Fisher test. If the two experimental arms are greater than the placebo arm, they will be compared to each other at risk 0.05.
  - Cumulative incidence of mechanical ventilation on D28: The cumulative incidence estimates will be made using the gray method and compared according to the previous procedure using a gray test.
  - The duration of the resuscitation stay: Estimates of the median length of stay in intensive care will be made from an inverted Kaplan Meier estimator and compared according to the previous procedure using a log rank test.

#### Calculation Hypotheses for the Number of Subjects Required and Result

The MDP-A1 subscale of the Multidimensional Dyspnea Profile is the main evaluation criterion of this study. This subscale is similar to the visual analog scale. Assuming a difference of 1 (on the primary efficacy endpoint, superiority study) between one of the 2 experimental groups and the placebo group and a standard deviation of 1, with a first species risk (alpha risk) of 2.5% (to take into account the 2 comparisons of each experimental group with placebo) and a power of 80% (beta risk at 20%), it will be necessary to include 21 patients per group or 63 patients in total. This number is consistent with that of other studies in tDCS (29, 60, 61).

The main analysis will be carried out according to the intention to treat principle. Only patients who have withdrawn their consent can be excluded from the analysis. If the period of inclusion in the research is still active, patients who have withdrawn their consent will be replaced. Patients who have decided to discontinue treatment planned in the trial, lost to follow-up or discontinued from the trial will be replaced, as will patients for whom there have been technical problems. Analyzes will be carried out with the intention of treating. Regarding missing data issue, in case of patient drop-out, in order to clearly understanding of the effectiveness of the therapy, we will first report results based on the completed cases, then secondarily with mixed-model or similar approaches which take into account partially available data, and finally with multiple imputation techniques.

## Ethical and Legal Aspects

This study was approved by our legal ethical committee: Comité de Protection des Personnes Ouest III, Université de Poitiers; CPP number 170946 and renewed on april 28th 2020. Informed consent should be obtained from each patient or family member before inclusion in the study.

## Conclusions

Dyspnea is a painful suffering that often reaches unbearable limits. Unfortunately, it is very frequent in intensive care and constitutes a major factor affecting the prognosis of intensive care patients, and more particularly patients under mechanical ventilation. Many COVID-19 patients continue to suffer from it, despite being put on mechanical ventilation and the use of relaxing and analgesic drugs (40). The effectiveness of the treatments currently available therefore remains very limited and there is a pressing need to develop other innovative treatments, including non- medicinal ones, in order to combat this scourge even more effectively and reduce the suffering of patients (39). The tDCS has demonstrated efficacy in pain relief, which shares the same neural substrates as dyspnea. It is a painless, easy to use and non-invasive technique. The originality and the innovative character of this study reside in the development of an effective method of treatment by neuro-modulation non-invasive and easy to use to combat this respiratory suffering in COVID-19 patient. Effective relief of dyspnea with tDCS would also have a significant impact on the prognosis of these patients. Finally, one may argue that it could have been better to conduct a multisession tDCS study, however this study is a pilot, designed to assess whether a single 30 min tDCS session could be beneficial for dyspnea relief in this specific patients' population. According to the findings of the present study we will conceive and assess outcome of other tDCS treatment strategies and designs including multisession ones.

## Author Contributions

EA, FL, DA, and TS developed the study concept. All authors wrote and drafted the manuscript, read, and approved the final manuscript.

## Conflict of Interest

The authors declare that the research was conducted in the absence of any commercial or financial relationships that could be construed as a potential conflict of interest.
